# Investigation of the correlation between radiomorphometric indices in cone-beam computed tomography images and dual X-ray absorptiometry bone density test results in postmenopausal women

**DOI:** 10.1186/s12880-025-01739-5

**Published:** 2025-06-05

**Authors:** Sara Rafieizadeh, Sima Lari, Mohammad Mahdi Maleki, Abbas Shokri, Leili Tapak

**Affiliations:** 1 Dental Implants Research Centre, Department of Oral and Maxillofacial Radiology, Dental School, Hamadan University of Medical Sciences, Hamadan, Iran; 2 Department of Oral and Maxillofacial Radiology, Dental School, Shahid Beheshti University of Medical Sciences, Hamadan, Iran; 3 Student Research Committee, Hamadan, University of Medical Sciences, Hamadan, Iran; 4 Dental Implants Research Centre, Dental School, Hamadan University of Medical Sciences, Hamadan, Iran; 5 Department of Biostatistics, Hamadan University of Medical Sciences, Hamadan, Iran; 6 Dental School, Hamadan University of Medical Sciences, Shahid Fahmideh Blvd, Hamadan, 6516647447 Iran

**Keywords:** Cone-beam computed tomography (CBCT), Bone mineral density (BMD), Radiomorphometric indices, Osteoporosis screening, Postmenopausal womens

## Abstract

**Objective:**

Osteoporosis is a prevalent skeletal disorder characterized by reduced bone mineral density (BMD) and structural deterioration, resulting in increased fracture risk. Early diagnosis is crucial to prevent fractures and improve patient outcomes. This study investigates the diagnostic utility of morphometric and cortical indices derived from cone-beam computed tomography (CBCT) for identifying osteoporotic postmenopausal women who were candidates for dental implant therapy, with dual-energy X-ray absorptiometry (DXA) used as the reference standard.

**Materials and methods:**

This cross-sectional study included 71 postmenopausal women, aged 50–79 years, who underwent CBCT imaging at the Oral and Maxillofacial Radiology Department of Hamadan University of Medical Sciences between 2022 and 2024. Participants with systemic conditions affecting bone metabolism were excluded. The morphometric indices—Computed Tomography Mandibular Index (CTMI), Computed Tomography Index Superior (CTI(S)), Computed Tomography Index Inferior (CTI(I)), and Computed Tomography Cortical Index (CTCI)—were measured at the mental foramen and antegonial regions using OnDemand3D Dental software. Bone mineral density (BMD) was assessed by DXA scans of the lumbar spine and femoral neck. In addition to traditional statistical analyses (Pearson’s correlation and one-way ANOVA with LSD test), a multilayer perceptron (MLP) neural network model was employed to evaluate the diagnostic power of CBCT indices.

**Results:**

DXA results based on the femoral neck T-scores categorized 38 patients as normal, 32 as osteopenic, and one as osteoporotic, while lumbar spine T-scores identified 38 normal, 22 osteopenic, and 11 osteoporotic patients. Significant differences (*p* < 0.05) were observed in most CBCT-derived indices, with the CTMI index demonstrating the most marked variation, especially between normal and osteoporotic groups (*p* < 0.001). Moreover, significant positive correlations were found between the CBCT indices and DXA T-scores across the lumbar spine, femoral neck, and total hip regions. The neural network model achieved an overall diagnostic accuracy of 75%, with the highest predictive importance attributed to antegonial CTCI and CTMI indices.

**Conclusion:**

This study highlights the significant correlation between CBCT-derived radiomorphometric indices such as CTMI, CTI(S), CTI(I), and CTCI at the mental foramen and antegonial regions and bone mineral density (BMD) in postmenopausal women. CBCT, particularly the CTMI index in the antegonial region, offers a cost-effective, non-invasive method for early osteoporosis detection, providing a valuable alternative to traditional screening methods.

## Introduction

Osteoporosis is a systemic skeletal disorder characterized by reduced bone mineral density and microarchitectural deterioration, which results in increased bone fragility and a heightened risk of fractures. This condition is a significant public health concern worldwide, particularly affecting elderly and middle-aged women, with postmenopausal women being most vulnerable due to the decrease in estrogen levels [1–3]. Early detection of individuals with low bone density is imperative, given the significant social and economic impacts of osteoporotic fractures [4]. It is estimated that approximately 50% of women and 20% of men will experience osteoporotic fractures during their lifetime [5].

The rate of bone loss accelerates significantly in women during the postmenopausal period—especially within the first 5–10 years—and continues thereafter [6]. Early diagnosis and fracture risk assessment have become increasingly important in light of available treatments that can modify the progression of osteoporosis [7]. Although BMD assessment via dual-energy X-ray absorptiometry (DXA) is considered the gold standard for osteoporosis diagnosis, its high cost limits its routine use [8].

Various imaging modalities, including quantitative computed tomography (QCT), quantitative ultrasonography (QUS), and cone-beam computed tomography (CBCT), have been utilized to evaluate BMD [9]. Since the bones in the oral cavity share similar structural and physiological characteristics with other skeletal bones, several studies have examined whether changes in mandibular bone can reflect systemic osteoporosis [10]. Dental radiographs, which are commonly used, may serve as convenient screening tools for osteoporosis during routine dental examinations [11].

Several methods exist for evaluating alveolar and cortical bone to predict low BMD [12, 13]. Previous research has suggested that mandibular radiomorphometric indices on conventional two-dimensional radiographs may serve as potential indicators of osteoporosis [14, 15]. Recent studies have underscored the potential of panoramic radiographs for identifying postmenopausal women with low BMD. For instance, Taguchi et al. emphasized that postmenopausal women exhibiting a severely eroded mandibular cortex on panoramic radiographs are at an increased risk of low skeletal BMD and fragility fractures, noting that a mandibular cortical width of less than 3 mm may indicate low BMD, even in the absence of fragility fractures [16]. Additionally, a systematic review and meta-analysis by Heuchert and Kozieł assessed the diagnostic accuracy of mandibular radiomorphometric indices in panoramic images for preliminary diagnosis of osteopenia and osteoporosis, suggesting that these indices can serve as valuable screening tools [17]. These studies underscore the importance of evaluating mandibular cortical structures in panoramic radiographs as a non-invasive and cost-effective approach to early osteoporosis detection in postmenopausal women. However, definitive indices using CBCT images have not been well established, despite CBCT’s ability to provide high-resolution, three-dimensional images without overlap or distortion [18].

Considering the high cost of DXA testing and the increasing number of elderly edentulous patients requiring implant therapy—where adequate bone quality and density are critical—the use of CBCT images to assess skeletal status without additional financial burden is advantageous. Moreover, prior studies have emphasized the utility of CBCT in analyzing trabecular bone structure at implant sites, and recent research has highlighted the potential of CBCT as a reliable tool for evaluating BMD [19, 20].

This study investigates the diagnostic utility of morphometric and cortical indices derived from cone-beam computed tomography (CBCT) such as CTMI, CTI(S), CTI(I) and CTCI for identifying osteoporotic postmenopausal women, using dual-energy X-ray absorptiometry (DXA) as the reference standard.

## Materials and methods

### Study population

We conducted a sample size calculation for correlational studies using an estimated r of 0.35, Zα = 1.96, and Zβ = 0.8416. The calculated sample size was 64, and we accounted for a 10% attrition rate to reach a final sample size of 71. We also provided references [21] supporting the natural imbalance observed in clinical populations.

This cross-sectional study included 71 postmenopausal women, aged 50–79 years, who presented to the Oral and Maxillofacial Radiology Department at Hamadan University of Medical Sciences between 2022 and 2024 for CBCT imaging. Inclusion criteria were edentulous women over 50 years of age, confirmed postmenopausal status, and the absence of systemic diseases affecting bone metabolism who patients who were candidates for implant therapy. Exclusion criteria encompassed conditions known to influence bone metabolism (e.g., cancer, osteomalacia, hyperthyroidism, hyperparathyroidism, severe renal or hepatic insufficiency, rheumatoid arthritis), a history of non-traumatic fractures, oophorectomy, the use of medications affecting bone turnover (such as corticosteroids or hormone therapy), and the consumption of tobacco or alcohol [4]. Participants were selected consecutively without discretionary selection to minimize potential selection bias.

### Imaging protocol

CBCT scans were obtained using the Cranex 3D system (Soredex, Tuusula, Finland) with the following parameters: 90 kVp, 10 mA, an exposure time of 14.6 s, a voxel size of 200 μm, a slice thickness of 0.5 mm, and a slice interval of 1 mm. To standardize measurements, all images were exported in DICOM format and analyzed using OnDemand3D Dental software (CyberMed, Seoul, Korea). Radiomorphometric indices—CTMI, CTI(S), CTI(I), and CTCI—were measured bilaterally at the mental foramen and antegonial regions. A random subset of 20 images was independently measured by two experienced oral radiologists to assess reliability. Both inter- and intra-observer reliability were evaluated using the intraclass correlation coefficient (ICC), with all ICC values exceeding 0.85, indicating excellent agreement.

### Measurement of morphometric indices (Fig. 1)

**Fig. 1:**
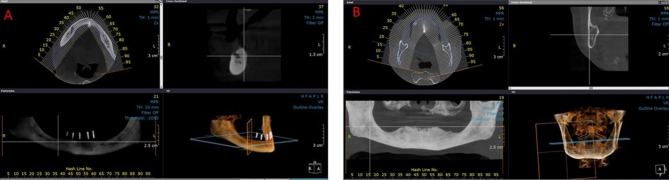
Selection of the mental foramen (**A**) and antegonial (**B**) regions and creation of cross-sectional images

**Fig. 2:**
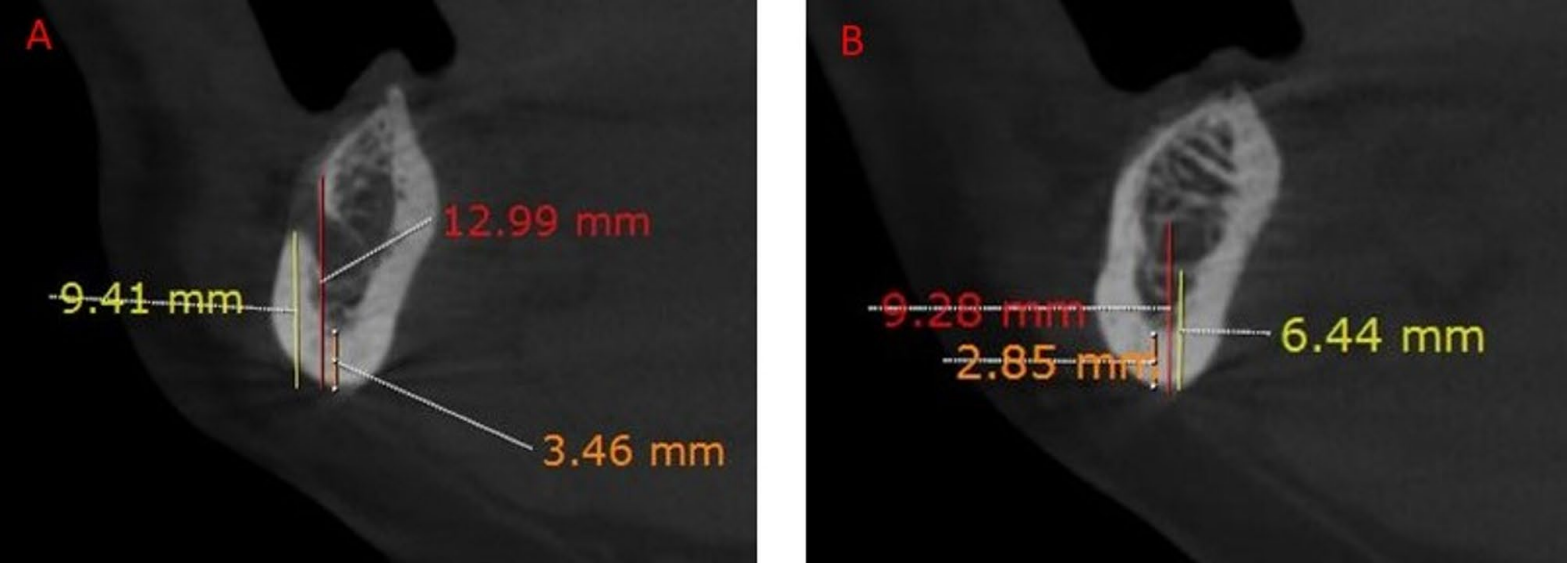
Measurement of CTMI (Orange label), CTI(S) (red label), and CTI(I) (yellow label) at the mental foramen (**A**) and antegonial (**B**) regions

**Fig. 3:**
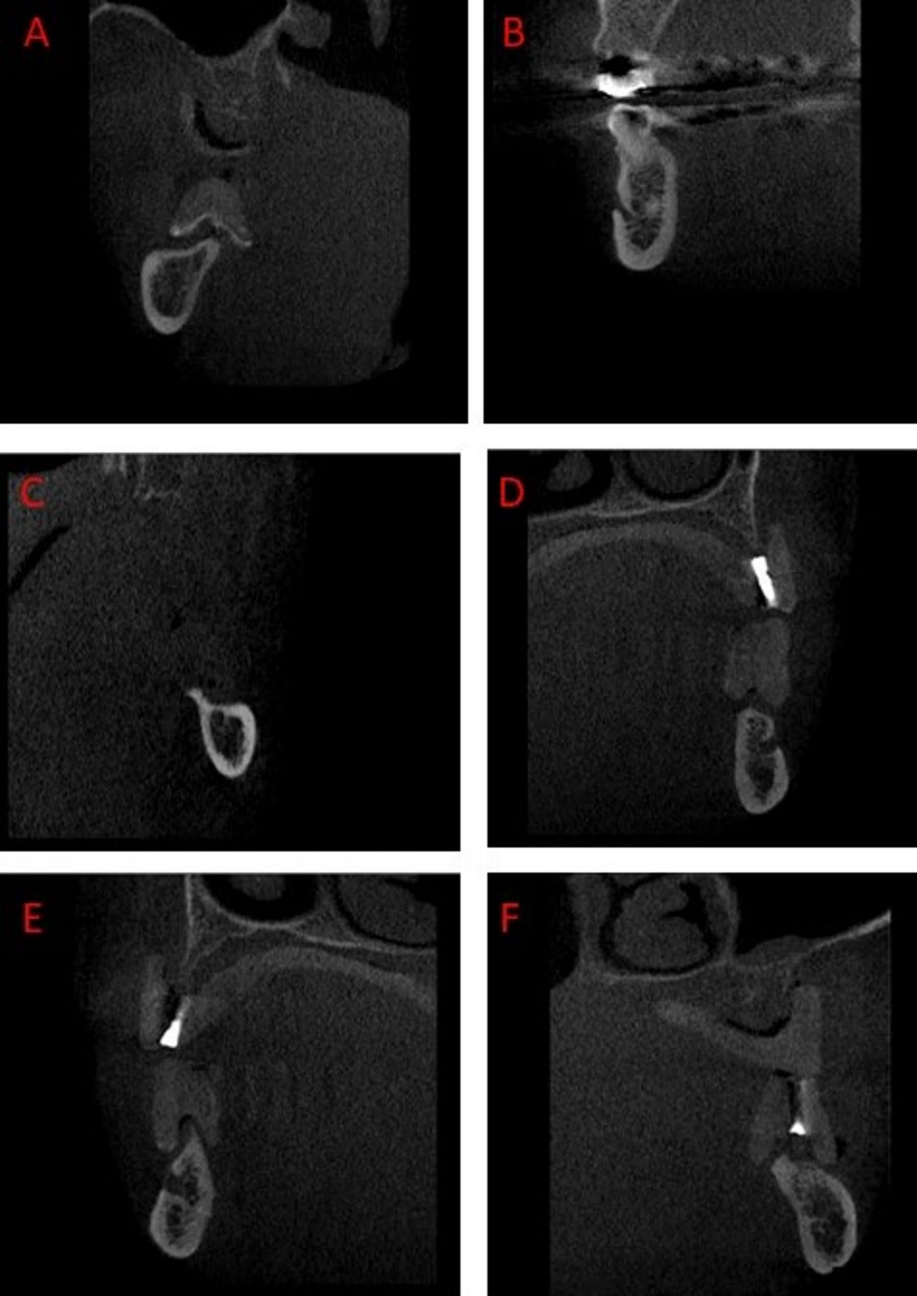
Examples of C1, C2, and C3 classifications at the mental foramen and antegonial regions. **A**: C1 at mental foramen, **B**: C2 at mental foramen, **C**: C3 at mental foramen, **D**: C1 at antegonial region, **E**: C2 at antegonial region, **F**: C3 at antegonial region

- **CTMI (Computed Tomography Mandibular Index)**: The cortical width at the inferior border of the mandible was measured at the mental foramen and antegonial regions using the Ruler tool in the software (Fig. 2).

- **CTI(S) (Computed Tomography Index Superior)**: The distance from the superior margin of the mental foramen to the inferior border of the mandible was measured and divided by the cortical width (Fig. 2).
- **CTI(I) (Computed Tomography Index Inferior)**: The distance from the inferior margin of the mental foramen to the inferior border of the mandible was measured and divided by the cortical width (Fig. 2) [22].
- **CTCI (Computed Tomography Cortical Index)**: The endosteal margin of the mandibular cortex was assessed and classified into three categories—C1 (normal cortex), C2 (moderately eroded cortex), and C3 (severely eroded cortex)—based on Klemetti’s classification [23](Figs. 3).

All measurements were performed independently by two calibrated observers experienced with the OnDemand3D Dental software.

### Bone mineral density assessment

Participants underwent DXA scanning of the lumbar spine and proximal femur using the OSTEOCORE device (France, 2007) with settings of 86 kVp, 9 mA, and a patient dose of 1 µGy/scan/h. BMD was expressed as T-scores, which represent the number of standard deviations from the mean peak bone mass of a young, healthy reference population. The classifications were as follows:

- **Normal**: T-score ≥ -1.
- **Osteopenia**: T-score between − 1 and − 2.5.
- **Osteoporosis**: T-score ≤ -2.5.

The DXA device was calibrated daily following the International Society for Clinical Densitometry (ISCD) guidelines, and all scans were performed by experienced technicians with over five years of expertise in bone densitometry.

The research hypotheses are defined as follows:

- **Null Hypothesis (H₀)**: There is no significant correlation between the radiomorphometric indices derived from CBCT images (such as CTMI, CTI(S), CTI(I), and CTCI) and the T-scores obtained from DXA in postmenopausal women.
- **Alternative Hypothesis (H₁)**: There is a significant correlation between the radiomorphometric indices from CBCT images and the DXA T-scores in postmenopausal women, such that a decrease in the radiomorphometric indices is associated with a decrease in bone mineral density (i.e., lower T-scores).

These hypotheses have been formulated based on the primary objective of the study—to evaluate the correlation between CBCT-derived imaging indices and DXA-assessed bone mineral density—thereby providing the necessary scientific framework for statistical analysis and interpretation of the results.

### Statistical analysis

Data were analyzed using SPSS software (version 25.0). Descriptive statistics (sample size, mean, standard deviation, minimum, and maximum values) were computed for eight variables across the three diagnostic groups (normal, osteopenic, and osteoporotic). We evaluated our statistical analyses and performed normality tests (using the Shapiro-Wilk test) on our continuous variables. The results confirmed that the data were normally distributed, which justified our use of parametric tests. Differences among these groups were assessed using one-way ANOVA, with corresponding *p*-values reported for each variable. For multiple comparisons, the Least Significant Difference (LSD) test was applied to evaluate pairwise differences between diagnostic groups. Notably, the normal and osteoporotic groups consistently demonstrated the largest mean differences, with highly significant differences (*p* < 0.001) observed across all variables. Furthermore, Pearson’s correlation test was used to examine the relationships between CBCT-derived radiomorphometric indices (CTMI, CTI(I), and CTI(S))—measured at both the mental foramen and antegonial regions—and T-scores from DXA assessments of the lumbar vertebrae, femoral neck, and total hip. These statistical methods were chosen to robustly evaluate both group differences and the associations between continuous variables, thereby addressing the study’s objectives with appropriate rigor.

In addition to the classical correlation analysis, we employed a multilayer perceptron (MLP) model to evaluate the diagnostic power of the selected variables in classifying cases into Osteopenia, Osteoporosis, and normal categories. The dataset was randomly split into training (70%) and testing (30%) subsets.

### Neural network architecture

The neural network consisted of an input layer, a single hidden layer, and an output layer. The input layer incorporated nine standardized covariates: mental foramen CTMI, CTI(I), CTI(S), CTCI; antegonial CTMI, CTI(I), CTI(S), CTCI; and age. The hidden layer comprised four neurons and utilized the hyperbolic tangent (tanh) activation function to model non-linear patterns in the data. The output layer included three neurons corresponding to the diagnostic categories and employed the SoftMax activation function to produce class probability distributions. Model performance was assessed using the cross-entropy loss function, which measures the divergence between predicted and actual class distributions. All analyses were adjusted for age.

## Results

In this study, 71 women aged between 50 and 79 years, with a mean age of 55, were evaluated based on inclusion and exclusion criteria. Intra- and inter-observer agreement in assessing the indices were 91% and 87%, respectively. Patients were categorized into three groups: normal, osteopenic, and osteoporotic, as presented in Table 1.

**Table 1:** Classification of patients into three groups: normal/osteopenia/osteoporosis, based on the T-score results of femoral neck and lumbar spine

	T-score results of femoral neck		T-score results of lumbar spine	
	Frequency	Percentage	Frequency	Percentage
Osteoporosis	1	1.4	11	15.5
Osteopenia	32	45.1	22	31
Normal	38	53.5	38	53.5
Total	71	100	71	100

Based on the bone density test results summarized in Table 1, out of 71 patients, according to the femoral neck T-score, 38 were normal, 32 were osteopenic, and one was osteoporotic. According to the lumbar vertebrae T-score, 38 were normal, 22 were osteopenic, and 11 were osteoporotic. Descriptive findings of the bone density test are presented in Table 2.

**Table 2:** Descriptive analysis of the results obtained from bone density test (DEXA) in the lumbar vertebrae and femoral neck regions

	Number	Mean	Standard Deviation	Minimum	Maximum
Femoral Neck T-score	71	-0.676	1.307	-2.5	3.6
Femoral Neck Z-score	71	0.248	1.165	-1.8	3.7
Femoral Neck BMD	71	0.818	0.164	0.585	1.332
Lumbar Vertebrae T-score	71	0.752	1.638	-4.1	3.9
Lumbar Vertebrae Z-score	71	0.322	1.779	-3.3	4.6
Lumbar Vertebrae BMD	71	1.018	0.205	0.598	1.588

Measurements of indices CTI(I), CTI(S), and CTCI in the mental foramen and antegonial regions in CBCT images were performed for each patient. Descriptive findings, including mean, standard deviation, and minimum and maximum values of these indices in the three groups—normal, osteopenic, and osteoporotic—are presented in Table 3. Statistically significant differences between groups were assessed using ANOVA, with *p*-values reported for each variable. The results indicate significant differences (*p* < 0.05) in most variables, especially between the normal and osteoporotic groups. Variables like CTMI Index in antegonial and CTMI Index in mental foramen exhibited the largest differences. These findings highlight distinct structural changes among the diagnostic groups, emphasizing the progressive impact of bone density reduction. Additionally, the assessment of these indices in the two groups—normal and abnormal—is provided in Table 3.

**Table 3:** This table summarizes the descriptive statistics for eight variables across three diagnostic groups (normal, osteopenic, and osteoporotic), including the sample size (N), mean, standard deviation, minimum, and maximum values. Statistically significant differences between groups were assessed using ANOVA, with *p*-values reported for each variable. The results indicate significant differences (*p* < 0.05) in most variables, especially between the normal and osteoporotic groups. Variables like CTMI index in antegonial and CTMI index in mental foramen exhibited the largest differences. These findings highlight distinct structural changes among the diagnostic groups, emphasizing the progressive impact of bone density reduction

		CTMI Index in Mental Foramen (mm)	CTI(I) Index in Mental Foramen (mm)	CTI(S) Index in Mental Foramen (mm)	CTCI Index in Mental Foramen	CTMI Index in Antegonial (mm)	CTI(I) Index in Antegonial (mm)	CTI(S) Index in Antegonial (mm)	CTCI Index in Antegonial
P – value		< 0.001	0.002	0.001	< 0.001	< 0.001	< 0.001	< 0.001	< 0.001
Normal	**Mean**	3.51	0.286	0.243	1.35	3.54	0.513	0.352	1.17
	**Standard Deviation**	0.734	0.058	0.048	0.668	0.729	0.143	0.081	0.380
	**Minimum**	1.86	0.165	0.137	1	2.20	0.266	0.198	1
	**Maximum**	4.900	0.422	0.335	3	5.200	1.00	0.521	2
Osteopenia	**Mean**	3.210	0.259	0.212	2.06	3.120	0.427	0.299	1.62
	Standard Deviation	0.747	0.059	0.053	0.689	0.604	0.103	0.069	0.519
	**Minimum**	1.80	0.158	0.121	1	2.020	0.241	0.180	1
	**Maximum**	4.900	0.395	0.350	3	4.740	0.740	0.481	3
Osteoporosis	**Mean**	2.530	0.232	0.182	2.21	2.600	0.400	0.284	1.91
	**Standard Deviation**	0.984	0.070	0.062	0.721	0.654	0.102	0.070	0.515
	**Minimum**	1.16	0.101	0.077	1	1.390	0.217	0.155	1
	**Maximum**	4.70	0.388	0.326	3	3.400	0.629	0.485	3
Abnormal	**Mean**	3.030	0.252	0.205	2.10	2.980	0.420	0.295	1.70
	**Standard Deviation**	0.865	0.063	0.564	0.696	0.655	0.103	0.069	0.531
	**Minimum**	1.160	0.101	0.077	1	1.39	0.217	0.155	1
	**Maximum**	4.900	0.395	0.350	3	4.740	0.740	0.485	3
Total	**Mean**	3.200	0.263	0.215	1.84	3.170	0.452	0.315	1.51
	**Standard Deviation**	0.851	0.063	0.055	0.771	0.729	0.126	0.078	0.544
	**Minimum**	1.160	0.101	0.077	1	1.390	0.217	0.155	1
	**Maximum**	4.900	0.422	0.350	3	5.200	1.000	0.521	3

Table 4 presents the results of multiple comparisons using the Least Significant Difference (LSD) test to evaluate differences between diagnostic groups (normal, osteopenic, and osteoporotic) across various variables. Significant differences were observed in most comparisons, particularly between the normal and osteoporotic groups, which consistently showed the largest mean differences with highly significant *p*-values (*p* < 0.001) across all variables. Notably, variables such as CTMI Index in mental foramen, CTI(S) Index in mental foramen, and CTMI Index in antegonial demonstrated significant differences between osteopenic and osteoporotic groups as well. However, some comparisons, like CTI(I) Index in mental foramen and CTI(I) Index in antegonial between osteopenic and osteoporotic groups, did not reach statistical significance (*p* > 0.05). These findings highlight the progressive nature of bone changes across the diagnostic categories, emphasizing the distinct characteristics of osteoporotic conditions.

**Table 4:** The table presents the results of multiple comparisons using the least significant difference (LSD) test to evaluate differences between diagnostic groups (normal, osteopenic, and osteoporotic) across various variables. significant differences were observed in most comparisons, particularly between the normal and osteoporotic groups, which consistently showed the largest mean differences with highly significant *p*-values (*p* < 0.001) across all variables. Notably, variables such as CTMI index in mental foramen, CTI(S) index in mental foramen, and CTMI index in antigonial demonstrated significant differences between osteopenic and osteoporotic groups as well. However, some comparisons, like CTI(I) index in mental foramen and CTI(I) index in antigonial between osteopenic and osteoporotic groups, did not reach statistical significance (*p* > 0.05). These findings highlight the progressive nature of bone changes across the diagnostic categories, emphasizing the distinct characteristics of osteoporotic conditions

Variable	Group Comparison	Mean Difference	Std. Error	*P*-Value
CTMI Index in Mental Foramen (mm)	Normal vs. Osteopenia	0.29944	0.29944	0.29944
	Normal vs. Osteoporosis	0.98237	0.98237	0.98237
	Osteopenia vs. Osteoporosis	0.68293	0.68293	0.68293
CTI(I) Index in Mental Foramen (mm)	Normal vs. Osteopenia	0.02672	0.02672	0.02672
	Normal vs. Osteoporosis	0.05343	0.05343	0.05343
	Osteopenia vs. Osteoporosis	0.02671	0.02671	0.02671
CTI(S) Index in Mental Foramen (mm)	Normal vs. Osteopenia	0.02159	0.02159	0.02159
	Normal vs. Osteoporosis	0.05142	0.05142	0.05142
	Osteopenia vs. Osteoporosis	0.02983	0.02983	0.02983
CTCI Index in Mental Foramen	Normal vs. Osteopenia	-0.705	-0.705	-0.705
	Normal vs. Osteoporosis	-0.854	-0.854	-0.854
	Osteopenia vs. Osteoporosis	-0.150	-0.150	-0.150
CTMI Index in Antigonial (mm)	Normal vs. Osteopenia	0.41818	0.41818	0.41818
	Normal vs. Osteoporosis	0.93543	0.93543	0.93543
	Osteopenia vs. Osteoporosis	0.51726	0.51726	0.51726
CTI(I) Index in Antigonial (mm)	Normal vs. Osteopenia	0.08575	0.08575	0.08575
	Normal vs. Osteoporosis	0.11233	0.11233	0.11233
	Osteopenia vs. Osteoporosis	0.02658	0.02658	0.02658
CTI(S) Index in Antigonial (mm)	Normal vs. Osteopenia	0.05345	0.05345	0.05345
	Normal vs. Osteoporosis	0.06806	0.06806	0.06806
	Osteopenia vs. Osteoporosis	0.01460	0.01460	0.01460
CTCI Index in Antigonial	Normal vs. Osteopenia	-0.451	-0.451	-0.451
	Normal vs. Osteoporosis	-0.743	-0.743	-0.743
	Osteopenia vs. Osteoporosis	-0.292	-0.292	-0.292

The correlation between the indices CTMI, CTI(I), and CTI(S) in the mental foramen and T-scores obtained from the bone density test is shown in Table 5, indicating a significant positive correlation between lumbar vertebrae and femoral neck T-scores, total hip, and the indices CTMI, CTI(I), and CTI(S) in the mental foramen.

**Table 5:** Pearson correlation test between CTMI, CTI(I), CTI(S) mental foramen and antigonial indices and t-scores of lumbar vertebrae, femoral neck, and total hip

		T-Score of Lumbar Vertebrae	T-Score of Femoral Neck	T-Score of Total Hip
CTMI Index in Mental Foramen	Pearson Correlation Significance (Sig.)	0.470 0.000	0.245 0.004	0.234 0.006
CTI(I) Index in Mental Foramen	Pearson Correlation Significance (Sig.)	0.356 0.000	0.180 0.035	0.225 0.008
CTI(S) Index in Mental Foramen	Pearson Correlation Significance (Sig.)	0.397 0.000	0.207 0.015	0.226 0.008
CTMI Index in Antigonial	Pearson Correlation Significance (Sig.)	0.464 0.000	0.351 0.000	0.301 0.000
CTI(I) Index in Antigonial	Pearson Correlation Significance (Sig.)	0.273 0.002	0.303 0.000	0.393 0.000
CTI(S) Index in Antigonial	Pearson Correlation Significance (Sig.)	0.289 0.000	0.319 0.000	0.379 0.001

Similarly, the correlation between the indices CTMI, CTI(I), and CTI(S) in the antegonial region and T-scores is presented in Table 5, showing a significant positive correlation between lumbar vertebrae and femoral neck T-scores, total hip, and these indices in the antegonial region.

The classification results for diagnosing the three categories were as follows: the sensitivity for identifying normal, osteopenia, and osteoporosis cases was 62.5%, 95.5%, and 33.3%, respectively; the specificity for diagnosing these categories was 95.8%, 75%, and 100%, respectively; and the overall diagnostic accuracy across all three categories was 75%.

**Fig. 4:**
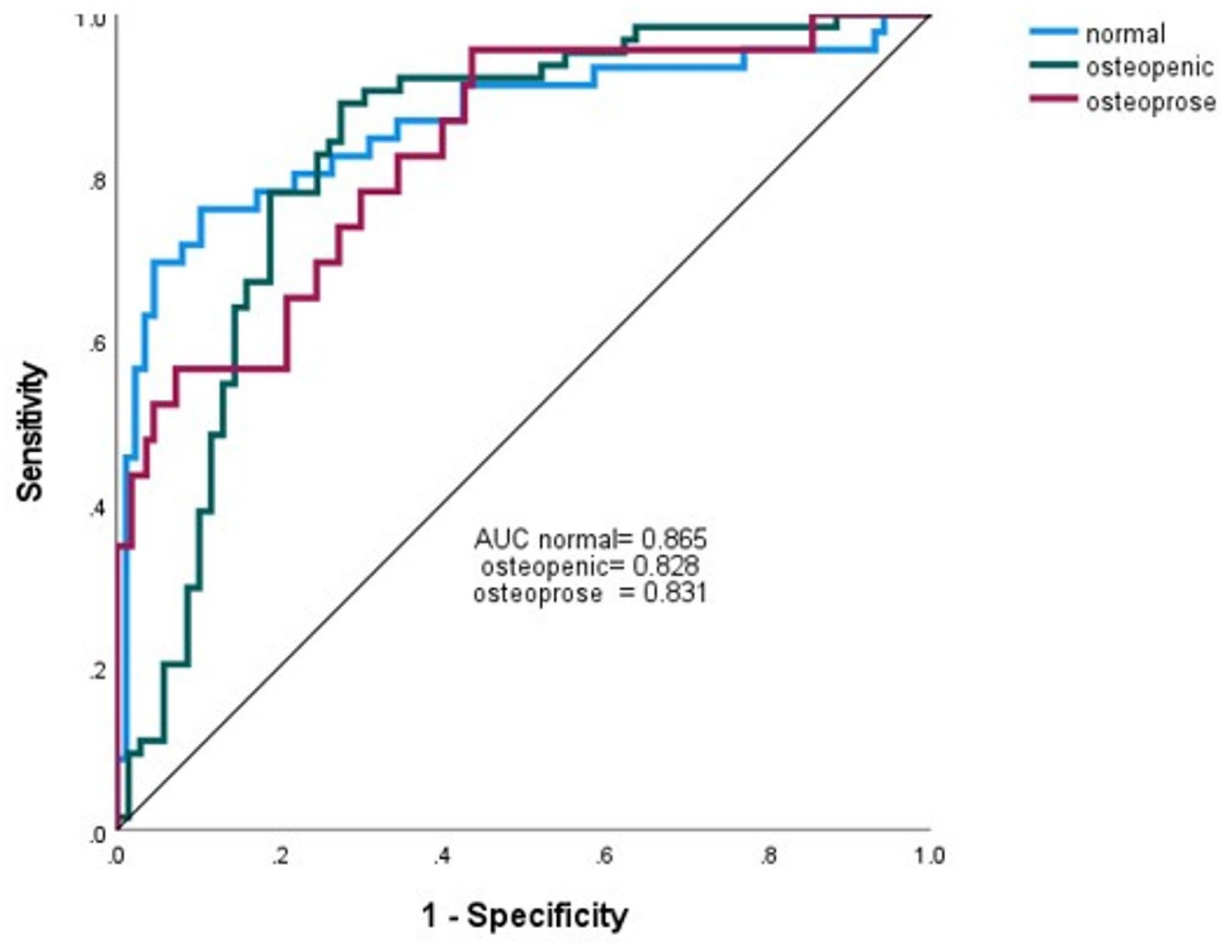
Area under the ROC curve obtained from the multilayer perceptron artificial neural network analysis for diagnosing normal, osteopenia, and osteoporosis cases

Figure 4 presents the ROC curves generated by the multilayer perceptron artificial neural network analysis. As shown, all AUC values exceed 0.7, indicating strong diagnostic performance of the model.

**Fig. 5:**
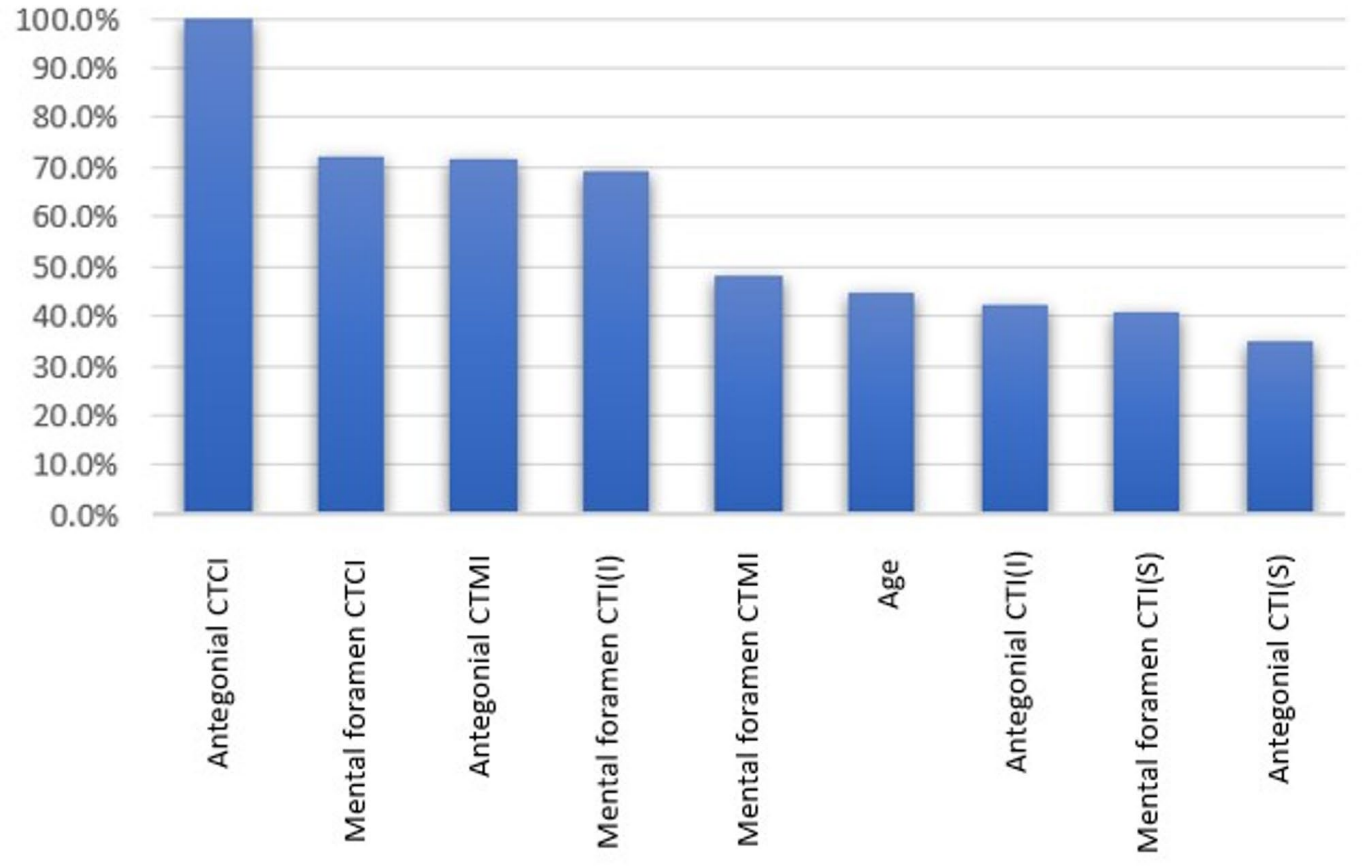
Variable importance chart derived from the multilayer perceptron artificial neural network analysis for diagnosing normal, osteopenia, and osteoporosis cases

Figure 5 illustrates the variable importance scores derived from the multilayer perceptron analysis in classifying normal, osteopenia, and osteoporosis cases. According to the results, the four most important variables for distinguishing between normal and abnormal cases were antegonial CTCI, mental foramen CTCI, antegonial CTMI, and mental foramen CTI(I), respectively.

## Discussion

One of the significant health issues affecting most women aged 50 and over is osteoporosis due to decreased estrogen levels post-menopause. Early detection of osteoporosis is crucial to increase bone mass retention, reduce the risk of fractures in the hip, vertebrae, and other bones, and prevent subsequent pain [24–26]. Given the considerable percentage of patients with bone density disorders among women over 50, using appropriate screening methods for early identification of patients with low bone density seems necessary. Currently, the gold standard in diagnosing osteoporosis is based on bone density measured by the DXA test in the hip and spinal regions; however, as a routine examination for osteoporosis screening, it is quite costly [27].

According to previous studies, a diverse percentage of asymptomatic patients undergoing bone density screening tests were identified as osteoporotic or osteopenic [28, 29]. In our study, among 71 asymptomatic patients, based on T-score results, 46.5% were abnormal, meaning both osteopenic and osteoporotic individuals. However, osteopenia and osteoporosis have different numerical ranges according to T-score indices (osteoporosis ≤ -2.5 and − 2.5 < osteopenia < -1), since the clinical treatment approach for both groups is the same, we considered them as one group.

The findings of this study highlight the potential of CBCT-derived morphometric indices in assessing bone mineral density (BMD) among postmenopausal women. A significant correlation was observed between indices such as CTMI, CTI(S), and CTI(I) in both the mental foramen and antegonial regions with DXA-derived T-scores. These results suggest that CBCT may serve as an adjunctive tool for osteoporosis screening, aligning with prior research [22, 23].

The CTMI index in the mental foramen region represents the thickness of the mandibular cortex in an area that accurately reflects bone status and is examined in most studies [30, 31]. Our results align with those of Brasileiro et al., who concluded that CBCT images could be used to diagnose osteoporosis in postmenopausal women. They found that CTMI values in the mental foramen region were lower in osteoporotic patients based on DXA results from lumbar vertebrae and femoral neck compared to normal individuals, and these indices are highly effective in distinguishing normal from osteoporotic patients [1]. However, Koh and Kim stated that although the mean CTMI values in the mental foramen were lower in the osteoporosis group (2.33 mm) compared to the normal group (3.22 mm), there was no significant correlation between the two groups [22]. Differences in sample size and group categorization may explain this discrepancy.

Comparisons between normal, osteopenic, and osteoporotic groups revealed that CTMI showed the most significant differences across groups, particularly between osteopenic and osteoporotic individuals. This aligns with findings from prior studies that indicate cortical thickness as a reliable indicator of bone density status [25, 27]. However, the indices CTI(I) and CTI(S) did not exhibit significant differences between osteopenic and osteoporotic groups, suggesting that these indices may have lower sensitivity in distinguishing mild-to-moderate bone loss, similar to findings reported by Klemetti et al. [23].

Furthermore, our data indicate that measurements obtained from the antegonial region tend to display more pronounced differences between normal and osteoporotic groups. This may be attributable to more marked cortical changes in this region in response to systemic bone loss. Previous studies have similarly observed stronger correlations between mandibular cortical width at the antegonial notch and osteoporosis [28, 29].

Studies by Devlin and Horner, which examined the CTMI index in the mental foramen in panoramic images, also reported significant differences in this index between normal and osteoporotic groups. According to these studies, individuals with a mandibular cortex width ≤ 3 mm in the mental foramen region should be referred for further osteoporosis assessment [32, 33].

The CTMI index in the antegonial region measures the thickness of the mandibular cortex anterior to the mandibular angle [34]. Akshita D and Asha V stated that the AI (CTMI in the antegonial region) index is not an acceptable index for diagnosing individuals with low BMD, and one of the existing problems in evaluating this index is determining the line that best fits the anterior border of the ramus [35]. According to studies by Leite et al. and Ledgerton et al., the AI (antegonial) index is a weak indicator for assessing osteoporosis in radiographic images, which contrasts with our results [34, 36]. However, Dutra V et al. [37] and Bajoria AA et al. [38] reported that the values of the antegonial index in the osteoporotic group were lower than in the normal group, and it is a useful method for identifying groups at risk of osteoporosis, supporting our findings [39]. Differences in ethnicity and sample size may explain these discrepancies [40].

Despite these promising findings, our study has several limitations. The modest sample size may limit the generalizability of our results, and while CBCT provides high-resolution imaging, it does not offer direct measures of bone mineral density. Future studies with larger, more diverse cohorts and further validation—potentially incorporating histomorphometric analysis—are warranted to substantiate these findings.

Consistent with the findings of Francisco et al., our study supports opportunistic CBCT scans for evaluating bone mineral density and fracture risk. This approach enhances the ability to monitor disease progression, facilitating the early detection of osteoporosis. By identifying bone density loss at an earlier stage, CBCT scans offer valuable insights, ultimately helping clinicians provide more effective, personalized patient care, thereby improving clinical outcomes in managing osteoporosis and fracture risk [41].

In summary, our study demonstrates that CBCT-derived morphometric indices, particularly the CTMI, have significant potential as non-invasive screening tools for osteoporosis in postmenopausal women. Given the widespread availability of CBCT in dental settings, integrating these indices into routine radiographic evaluations could facilitate early detection and timely intervention, ultimately improving patient outcomes. Future research should aim to refine these indices further and explore their applicability across diverse populations.

## Conclusion

This study demonstrates that CBCT-derived radiomorphometric indices (CTMI, CTI(I), and CTI(S))—measured at the mental foramen and antegonial regions—correlate significantly with bone mineral density as determined by DXA in postmenopausal women. Notably, the CTMI index in the antegonial region proved effective in distinguishing osteoporotic patients from normal individuals.

Given that these patients were already undergoing CBCT imaging for implant planning, our findings highlight a valuable opportunity for opportunistic osteoporosis screening without requiring additional procedures or radiation. CBCT may serve as a practical, accessible, and cost-effective adjunct tool in identifying individuals at risk of osteoporosis, particularly when reduced CTMI values are observed. Although CBCT appears promising in detecting advanced bone loss, its sensitivity for identifying milder bone loss associated with osteopenia is limited, so it should be used alongside other diagnostic methods.

Future research should aim to validate these findings in larger, more diverse populations and refine the use of CBCT-derived indices to develop a standardized clinical algorithm for the early detection and management of osteoporosis.

## Data Availability

The datasets used and/or analyzed during the current study are available from the corresponding author upon reasonable request.

## Abbreviations

CBCT: Cone-Beam Computed Tomography
DXA: Dual-energy X-ray Absorptiometry
BMD: Bone Mineral Density
CTMI: Computed Tomography Mandibular Index
CTI(S): Computed Tomography Index Superior
CTI(I): Computed Tomography Index Inferior
CTCI: Computed Tomography Cortical Index
MLP: Multilayer Perceptron
ICC: Intraclass Correlation Coefficient
ISCD: International Society for Clinical Densitometry
AUC: Area Under the Curve
LSD: Least Significant Difference
SPSS: Statistical Package for the Social Sciences
SD: Standard Deviation
